# Expression Profiling and Functional Analysis of Circular RNAs in Inner Mongolian Cashmere Goat Hair Follicles

**DOI:** 10.3389/fgene.2021.678825

**Published:** 2021-06-11

**Authors:** Fangzheng Shang, Yu Wang, Rong Ma, Zhengyang Di, Zhihong Wu, Erhan Hai, Youjun Rong, Jianfeng Pan, Lili Liang, Zhiying Wang, Ruijun Wang, Zhihong Liu, Yanhong Zhao, Zhixin Wang, Jinquan Li, Yanjun Zhang

**Affiliations:** ^1^College of Animal Science, Inner Mongolia Agricultural University, Hohhot, China; ^2^Key Laboratory of Mutton Sheep Genetics and Breeding, Ministry of Agriculture, Hohhot, China; ^3^Key Laboratory of Animal Genetics, Breeding and Reproduction, Hohhot, China; ^4^Engineering Research Center for Goat Genetics and Breeding, Hohhot, China

**Keywords:** circRNA, cashmere goat, hair follicles, expression profile, functional analysis

## Abstract

**Background:**

Inner Mongolian cashmere goats have hair of excellent quality and high economic value, and the skin hair follicle traits of cashmere goats have a direct and important effect on cashmere yield and quality. Circular RNA has been studied in a variety of tissues and cells.

**Result:**

In this study, high-throughput sequencing was used to obtain the expression profiles of circular RNA (circRNA) in the hair follicles of Inner Mongolian cashmere goats at different embryonic stages (45, 55, 65, and 75 days). A total of 21,784 circRNAs were identified. At the same time, the differentially expressed circRNA in the six comparison groups formed in the four stages were: d75vsd45, 59 upregulated and 33 downregulated DE circRNAs; d75vsd55, 61 upregulated and 102 downregulated DE circRNAs; d75vsd65, 32 upregulated and 33 downregulated DE circRNAs; d65vsd55, 67 upregulated and 169 downregulated DE circRNAs; d65vsd45, 96 upregulated and 63 downregulated DE circRNAs; and d55vsd45, 76 upregulated and 42 downregulated DE circRNAs. Six DE circRNA were randomly selected to verify the reliability of the sequencing results by quantitative RT-PCR. Subsequently, the circRNA corresponding host genes were analyzed by the Gene Ontology (GO) and the Kyoto Encyclopedia of Genes and Genomes (KEGG) pathway. The results showed that the biological processes related to hair follicle growth and development enriched by GO mainly included hair follicle morphogenesis and cell development, and the signaling pathways related to hair follicle development included the Notch signaling pathway and NF-κB signaling pathway. We combined the DE circRNA of d75vsd45 with miRNA and mRNA databases (unpublished) to construct the regulatory network of circRNA–miRNA–mRNA, and formed a total of 102 pairs of circRNA–miRNA and 126 pairs of miRNA–mRNA interactions. The binding relationship of circRNA3236–chi-miR-27b-3p and circRNA3236–chi-miR-16b-3p was further verified by dual-luciferase reporter assays, and the results showed that circRNA3236 and chi-miR-27b-3p, and circRNA3236 and chi-miR-16b-3p have a targeted binding relationship.

**Conclusion:**

To summarize, we established the expression profiling of circRNA in the fetal skin hair follicles of cashmere goats, and found that the host gene of circRNA may be involved in the development of hair follicles of cashmere goats. The regulatory network of circRNA–miRNA–mRNA was constructed and preliminarily verified using DE circRNAs.

## Background

There are two kinds of hair follicles in the skin of cashmere goats, namely, primary hair follicles and secondary hair follicles. Primary hair follicles produce coarse hairs, and secondary hair follicles produce cashmere. The structural characteristics of the skin and hair follicles of cashmere goats are not only important correlates of their biological characteristics but also have a direct and important effect on the yield and quality of cashmere. The hair follicle is a skin accessory organ with complex morphology and structure; it controls the growth of hair, and its most prominent feature is regeneration. The initiation of morphogenesis of primary and secondary hair follicles in cashmere goats occurs at different stages of embryonic development, and the initiation of primary hair follicles is earlier than that of secondary hair follicles. At the embryonic stage of 45–55 days, the skin forms a complete epidermal structure, and the hair follicles have not yet appeared; at 55–65 days, the primary hair follicles begin to develop in various parts of the fetus, and the keratinocytes in the basal layer of the epithelium are arranged in a fence to form hair buds, but the formation of primary hair follicles in the lateral part of the body is later than that in other parts (such as the top of the head, shoulder, and neck). At 65 days, obvious primary follicle hair buds are observed on the sides of the body. At 65–75 days, the primordial bodies of secondary hair follicles are observed in various parts of the fetus, and secondary hair follicles begin to occur and grow from the epidermis near the primary hair follicles. Similar to the primary hair follicles, the formation of secondary hair follicles in the lateral part of the body is later than that in other parts. At 75 days, obvious secondary hair follicle hair buds are observed on the side of the body (Supplementary Figure 1; Zhang et al., 2006, 2007).

The hair follicle traits of cashmere goats have a direct and important effect on the quantity and quality of cashmere. The ultimate goal of the research in the field of hair follicle growth and development in cashmere goats is to reveal the mechanism of cashmere growth and find the important genes related to cashmere growth. The morphogenesis and development of hair follicles may be related to some protein-coding genes. At present, it is considered that most of the signaling molecules regulating hair follicle morphogenesis belong to the Wnt pathway (Li et al., 2004), tumor necrosis factor (TNF) family, fibroblast growth factor (FGF) family (Milla, 2002), bone morphogenetic protein (BMP) family (Thomadakis et al., 1999), Sonic hedgehog (SHH) conduction pathway (McMahon et al., 2003), transforming growth factor (TGF) family (Ullrich and Paus, 2005), and NOTCH conduction pathway (Crowe et al., 1998). Some of these coding genes are stimulants of hair follicle development and some are inhibitors, which are repeatedly used to regulate each other.

miRNA is an early non-coding RNA in hair follicles. Researchers identified 22 new miRNAs and 316 conserved miRNAs in adult Inner Mongolia cashmere goats and speculated that miRNA-203 may play an important role in the growth of skin and hair follicles (Liu et al., 2012), and verified that miRNA-203 may regulate the development of cashmere goat hair follicles by targeting *DDOST* and *NAE1* (Ma et al., 2021). In recent years, the role of lncRNA in skin and hair follicles has been gradually a concern of researchers. Studies have found that lncRNA-000133 has a complex regulatory relationship with related miRNAs and their target genes. Overexpression of lncRNA-000133 leads to a significant increase in the relative expression of *ET-1*, *SCF*, *ALP*, and *LEF1* in dermal papilla cells (Zheng et al., 2019); lncRNA-599547 regulates the expression of *Wnt10b* gene by targeting miR-15b-5p, thus, inducing the differentiation of dermal papilla cells (Yin et al., 2020).

Circular RNA (circRNA) is a special kind of RNA, which has no free 5′ cap structure and 3′ poly (A) structure, and is insensitive to nuclease (William and Norman, 2014). CircRNAs can be divided into four types according to its origin: intron circRNA, exon circRNA, exon–intron circRNA, and intergenic circRNA (Chen and Li, 2015). The main mechanisms of action of circRNAs include regulating the expression of the host genes (Zhang et al., 2013; You et al., 2015), interacting with RNA-binding proteins (Xu et al., 2018); translating proteins (Conn et al., 2015); and acting as competitive endogenous RNA to regulate the expression of genes (Hansen et al., 2013; Memczak et al., 2013; Westholm et al., 2014). A current research focus is the action of circRNA, through competitive binding to miRNA, in regulating gene expression to complete the regulation of life activities that has become a research hotspot. Studies have shown that circLMO7 can enhance the *HDAC4* expression of the miR-378a-3p target gene through competitive binding of miR-378a-3p, promote muscle cell proliferation, and inhibit the differentiation of bovine myoblasts (Wei, 2017); CircARF3 adsorbs miR-103, to alleviate the targeted inhibition of miR-103 on *TRAF3* and alleviate adipose inflammation by promoting mitochondrial autophagy (Zhang, 2018). CircRNA3669 as competing endogenous RNA (ceRNA) adsorbs miR-26a and removes the downregulation of *RCN2* by miR-26a in dairy goat endometrial epithelial cells (Liu, 2019). However, circRNA studies on the regulation of hair follicle development in the embryonic stage of cashmere goats are still scarce.

In the previous research of our group, we analyzed the regulatory role of miRNA–mRNA in the development of hair follicles at embryo stage in cashmere goats (Han et al., 2020). In this study, in order to explore the pattern of expression and functional role of circRNAs in the development of fetal hair follicles of cashmere goats, we first used a high-throughput sequencing technique to construct the circRNA expression profiling of cashmere goats during the fetal period (45, 55, 65, and 75 days), and identified the DE circRNAs in different comparison groups. At the same time, the host genes of DE circRNAs were analyzed by Gene Ontology (GO) and the Kyoto Encyclopedia of Genes and Genomes (KEGG) pathway. Following this, the regulatory network of circRNA–miRNA–mRNA was constructed by combining miRNA and mRNA databases, and the binding of circRNA–miRNA was verified by dual-luciferase reporter assays. This study has laid a foundation for further exploration of the regulatory role of circRNAs as ceRNAs in the hair follicles of cashmere goats and has also provided a new direction for the study of hair follicles development.

## Materials and Methods

### Animals and Samples

In this experiment, 12 3-year-old ewes with good production performance, the same growth environment, and the same feed were selected for breeding in Inner Mongolia Jinlai Animal Husbandry Technology Co., Ltd. (Hohhot, Inner Mongolia), and the breeding time was recorded. The environment of the cashmere goat farm meets the relevant requirements of the experimental facilities in the Chinese national standard “Experimental Animal Environment and Facilities” (GB14925-2010). Health status, pathogenic microorganism infections, and zoonotic infections were monitored to ensure animal safety and all animal experiments were performed in accordance with the “Guidelines for Experimental Animals” of the Ministry of Science and Technology (Beijing, China). A total of 12 fetal skin samples were collected during the four periods of 45, 55, 65, and 75 days of gestation of goats, immediately treated with DPEC water, and placed in liquid nitrogen. The samples were then stored in a refrigerator at −80°C for RNA-seq and quantitative RT-PCR (qRT-PCR) tests. All fetal skin samples were collected in accordance with the International Guiding Principles for Biomedical Research Involving Animals and approved by the Special Committee on Scientific Research and Academic Ethics of Inner Mongolia Agricultural University, responsible for the approval of biomedical research ethics of Inner Mongolia Agricultural University [Approval No. (2020) 056]. No specific permissions were required for these activities, and no endangered or protected species were involved.

### RNA Library Construction and Sequencing

This study sequenced 12 samples of lateral skin of cashmere goats at 45, 55, 65, and 75 days of fetal period. Total RNA was isolated and purified using Trizol reagent (Invitrogen, Carlsbad, CA, United States), following the manufacturer’s procedure. The amount of RNA and purity of each sample were quantified using NanoDrop ND-1000 (NanoDrop, Wilmington, DE, United States). The RNA integrity was assessed using Agilent 2100. Approximately 5 μg of total RNA was used to deplete ribosomal RNA according to the instructions of the Ribo-Zero^TM^ rRNA Removal Kit (Illumina, San Diego, CA, United States), and the remaining RNA fragments were reverse transcribed using an RNA-seq Library Preparation Kit (Illumina) to form the final cDNA. Finally, we performed the paired-end sequencing on an Illumina Hiseq 4000 (LC Bio, Hangzhou, Zhejiang, China), following the vendor’s recommended protocol.

### Identification of Transcripts

According to the characteristics of circRNA structure and splicing sequence, and combined with literature reports, we used CIRCExploter2 (Zhang et al., 2014; Wang et al., 2020) and CIRI (Gao et al., 2015, 2018) to predict circRNAs, and integrate the results of the two software programs according to the starting and ending positions of circRNA. The following is the circRNA identification standard: (1) Mismatch ≤2; (2) back-spliced junctions reads ≥1; (3) two splice sites comprise less than 100 kb of the genome. According to the above identification and screening criteria, circRNA was identified more accurately.

### Differential Expression Analysis

We used SRPBM as a normalization method to quantify the expression of circRNA.

$\text{SRPBM}=\frac{\text{SR}\times 10^{9}}{N}$, where SR is the number of spliced reads, and *N* is the total number of mapped reads in the sample. Analysis of differentially expressed circRNA was done using R package-edge, differential multiples (fold change) and *P*-values were used by default to screen differential circRNAs; that is, genes that simultaneously satisfy the absolute value of log2 (fold change) greater than or equal to 1 and *P*-value less than or equal to 0.05 are marked as yes; otherwise, they are marked as no.

### Gene Ontology and Kyoto Encyclopedia of Genes and Genomes Pathway Enrichment Analysis

Gene Ontology and KEGG enrichment analysis is the use of all GO and KEGG annotated circRNA host genes. The gene ontology database^1^ was used to perform functional annotations on DE transcripts of three components, namely, biological processes (BPs), molecular function (MF), and cellular component (CC). Pathway significant enrichment analysis can identify that the host genes are involved in the major biochemical metabolic pathways and signal transduction pathways using the KEGG database^2^. The hypergeometric test was used to analyze GO enrichment of host genes and the statistical enrichment of host genes in KEGG pathways. GO terms and KEGG pathways (corrected *P*-value < 0.05) were considered significantly enriched.

### Construction of CircRNA Regulatory Networks

In this study, two software programs, Targetscan^3^ and miRanda^4^, were used to predict the miRNA targeted by circRNA. The target genes targeting miRNA were predicted, and the circRNA–miRNA–mRNA regulatory network was constructed preliminarily. Finally, Cytoscape (Shannon et al., 2003) was used to visualize it.

### qRT-PCR

In accordance with the manufacturer’s instructions, we used Trizol reagent (Takara, Dalian, Liaoning, China) to extract the total RNA from 12 skin samples representing the fetal periods of cashmere goats. Subsequently, we used a PrimeScript RT Reagent Kit with gDNA Eraser (Takara, Dalian, Liaoning, China) to reverse transcribe RNA to cDNA. Following this, each sample with three duplicates, was tested on a LightCycler^®^ 96 Real-Time PCR system (Roche, Basel, Switzerland) using TB GreenPremix Ex Taq II (Takara, Dalian, Liaoning, China). The qRT-PCR conditions were: 95°C for 30 s and then 40 cycles at 95°C for 10 s, 60°C for 30 s, followed by 72°C for 10 s. β-actin was used as the reference gene (Shen et al., 2020; Zhang et al., 2020). The validated primers used for RT-qPCR are listed in the Supplementary Table 1. The expression levels were calculated using the 2^–ΔΔ*CT*^ method (Schmittgen and Livak, 2008).

### Dual-Luciferase Reporter Assays

Chi-miR-27b-3p mimics and chi-miR-16a-3p mimics were synthesized by Hanbio Biotechnology Company (Shanghai, China). The miRNA mimics were transfected into HEK 293T cells using the LipoFiter transfection reagent according to the manufacturer’s instructions. The psiCHECK2-circRNA3236-WT construct was generated by inserting the circRNA3236 fragments containing the miRNA binding sequence into the psiCHECK-2 vector (Promega) at the 3′ end of the Renilla luciferase gene. The mutant psiCHECK2-circRNA3236-MUT construct was generated by mutating the miRNA-binding sequence to the complementary sequence using overlapping extension PCR. For circRNA3236 luciferase assays, the HEK 293T cells were transfected with miRNA mimics and either the psiCHECK2-circRNA3236-WT or mutated psiCHECK2-circRNA3236-Mut reporter plasmid. At 48 h post-transfection, luciferase activity was measured using a dual-luciferase reporter assay system (Promega) according to the manufacturer’s instructions. The relative luciferase activities were calculated by comparing the Firefly/Renilla luciferase activity ratio.

### Statistical Analysis

SPSS18.0 (Beijing, China) was used to calculate the Spearman correlation coefficient and dual-luciferase assay. Results are expressed as the mean ± SEM, and statistically significant differences between two means were analyzed using *t*-test. A value of *P* < 0.05 was considered statistically significant.

## Results

### Identification and Characterization of CircRNAs in Hair Follicles of Cashmere Goats

In order to explore the expression pattern of circRNA in fetal skin hair follicles of cashmere goats, in this study, high-throughput sequencing was performed at four stages of the fetal phase of Inner Mongolian cashmere goats (Albas type). First, we constructed 12 libraries that removed ribosomal RNA of cashmere goats during the fetal period, which were named d45_1, d45_2, d45_3, d55_1, d55_2, d55_3, d65_1, d65_2, d65_3, d75_1, d75_2, and d75_3, respectively. These libraries were applied to the IlluminaHiseq4000 platform for RNA sequencing, and 1,063,299,566 raw reads were obtained from the 12 libraries (Table 1).

**TABLE 1:** Data quality control statistics.

**Sample**	**Raw data**	**Valid data**			**Valid ratio (reads)**	**Q20 %**	**Q30 %**	**GC content %**
	**Read**	**Base**	**Read**	**Base**				
d45_1	83,377,840	12.51G	80,991,260	12.15G	97.14	99.97	98.33	46
d45_2	80,073,650	12.01G	77,655,516	11.65G	96.98	99.97	98.33	45.50
d45_3	91,499,974	13.72G	87,070,648	13.06G	95.16	99.98	98.41	46
d55_1	100,354,248	15.05G	96,199,486	14.43G	95.86	99.98	98.45	46.50
d55_2	91,779,488	13.77G	88,089,256	13.21G	95.98	99.98	98.53	47
d55_3	94,888,486	14.23G	91,438,886	13.72G	96.36	99.98	98.48	47
d65_1	90,684,330	13.60G	87,483,330	13.12G	96.47	99.97	98.17	47
d65_2	102,708,880	15.41G	99,047,498	14.86G	96.44	99.98	98.47	46.50
d65_3	80,732,910	12.11G	77,823,688	11.67G	96.40	99.97	98.34	47
d75_1	81,140,600	12.17G	77,983,860	11.70G	96.11	99.98	98.45	45
d75_2	83,141,742	12.47G	80,115,068	12.02G	96.36	99.98	98.46	45
d75_3	82,917,418	12.44G	79,990,864	12.00G	96.47	99.97	98.33	46

In these original reads, 1,023,889,360 effective reads were obtained by removing the reads with a connector (adaptor); the reads with N (N indicating that the base information cannot be determined) are greater than 5%, and those are of low quality (the bases with a quality value Q ≤ 10 accounting for more than 20% of the total reads) (Table 2). The Q20 (the proportion of bases with quality value ≥20, error rate <0.001) of each library was 99.90% and the Q30 (the proportion of bases with quality value ≥30, error rate <0.001) was above 98.1% (Table 1), indicating that the sequencing accuracy was high. Comparing the 1,023,889,360 effective reads with the reference genome, the percentage of the number of reads on the reference genome as a percentage of valid reads was more than 94%, the percentage of the number of reads compared with the unique location of the reference genome as a percentage of valid reads was more than 77%, and the number of reads compared with the multiple locations of the reference genome as a percentage of valid reads was more than 17%. Therefore, the utilization rate of the data was normal, and the original data obtained met the requirements of the subsequent circRNA analysis (Table 2) in terms of quantity and quality.

**TABLE 2:** Reference genome alignment read statistics.

**Sample**	**Valid reads**	**Mapped reads (%)**	**Unique mapped reads (%)**	**Multi mapped reads (%)**
d45_1	80,991,260	76,343,633 (94.26)	61,948,389 (76.49)	14,395,244 (17.77)
d45_2	77,655,516	74,877,365 (96.42)	61,031,456 (78.59)	13,845,909 (17.83)
d45_3	87,070,648	84,828,546 (97.42)	68,283,000 (78.42)	16,545,546 (19.00)
d55_1	96,199,486	93,762,003 (97.47)	76,182,223 (79.19)	17,579,780 (18.27)
d55_2	88,089,256	85,888,657 (97.50)	69,725,622 (79.15)	16,163,035 (18.35)
d55_3	91,438,886	89,149,678 (97.50)	71,925,770 (78.66)	17,223,908 (18.84)
d65_1	87,483,330	85,122,572 (97.30)	68,170,805 (77.92)	16,951,767 (19.38)
d65_2	99,047,498	96,402,904 (97.33)	79,282,917 (80.05)	17,119,987 (17.28)
d65_3	77,823,688	75,757,511 (97.35)	60,872,547 (78.22)	14,884,964 (19.13)
d75_1	77,983,860	75,996,848 (97.45)	62,127,737 (79.67)	13,869,111 (17.78)
d75_2	80,115,068	78,137,926 (97.53)	64,459,926 (80.46)	13,678,000 (17.07)
d75_3	79,990,864	77,918,087 (97.41)	63,626,735 (79.54)	14,291,352 (17.87)

**FIGURE 1:**
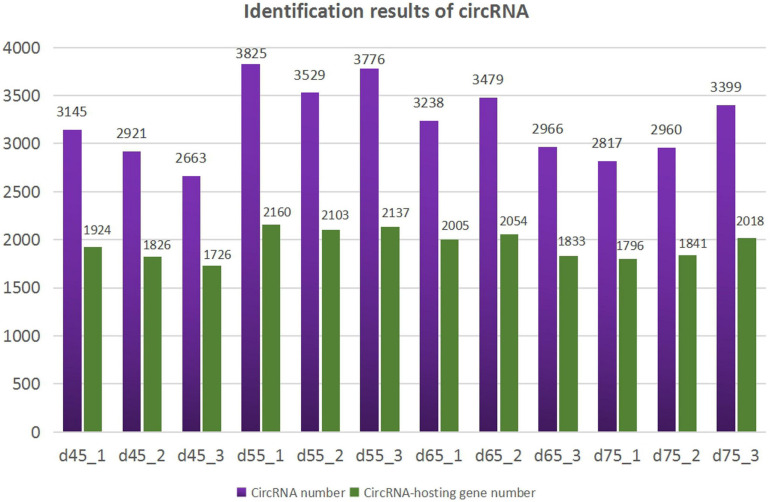
Identification results of circRNAs. The purple columns represent the number of circRNAs, and the green columns represent the number of circRNA-hosting genes.

**FIGURE 2:**
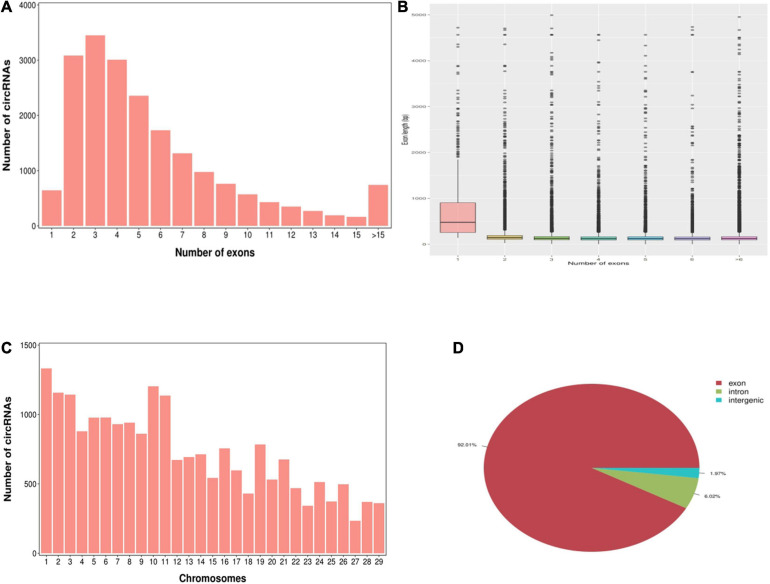
Characteristics of circRNAs in hair follicles of cashmere goats. **(A)** Distribution of the number of circRNAs per gene. The *x*-axis represents the number of circRNAs/host gene and the *y*-axis represents the number of circRNA. **(B)** Box plot showing the exon length of exon-derived circRNAs. The *x*-axis represents the number of exons that the circRNA contains and the y-axis represents the exon length. **(C)** Distribution of the identified circRNAs in each chromosome. The *x*-axis represents the number of chromosomes and the *y*-axis represents the number of circRNAs classified by different chromosomes. **(D)** Classification of circRNAs in hair follicles of cashmere goats.

According to the characteristics of the circRNA structure and splicing sequence, we used CIRCExploter2 and CIRI to predict circRNAs. The results showed that the total number of specific circRNA identified in each sample was more than 2,800, and the corresponding parental genes numbered more than 1,700 (Figure 1). Studies have found that most circRNAs contain two to four exons (Figure 2A). At the same time, the exon length of circRNAs indicates that the length of a single exon is longer than that of circRNAs composed of multiple exons (Figure 2B). Chromosome distribution analysis showed that circRNAs were distributed on almost all chromosomes, and the number of circRNAs on chromosomes 1, 10, and 11 was higher than those on other chromosomes (Figure 2C). Finally, exon circRNAs accounted for 92.01% (Figure 2D) of all circRNAs in the cashmere goat skin hair follicles.

### Analysis of Differences in CircRNAs

**FIGURE 3:**
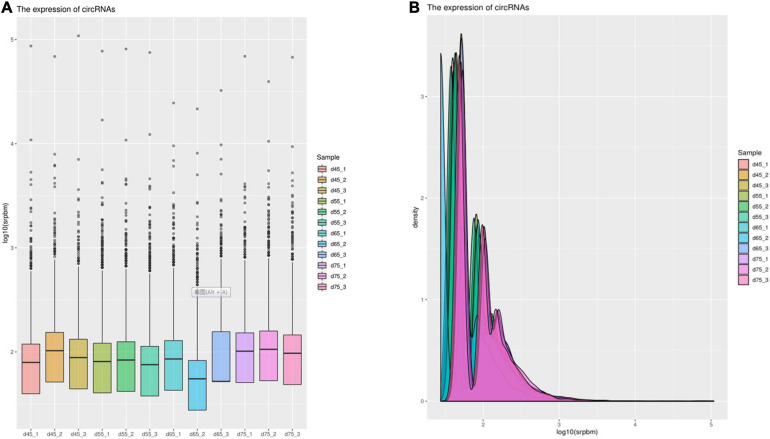
The expression of circRNAs. **(A)** Box plot showing the expression abundance of circRNAs in each sample. **(B)** Density plot of the expression density distribution of circRNAs in each sample.

**FIGURE 4:**
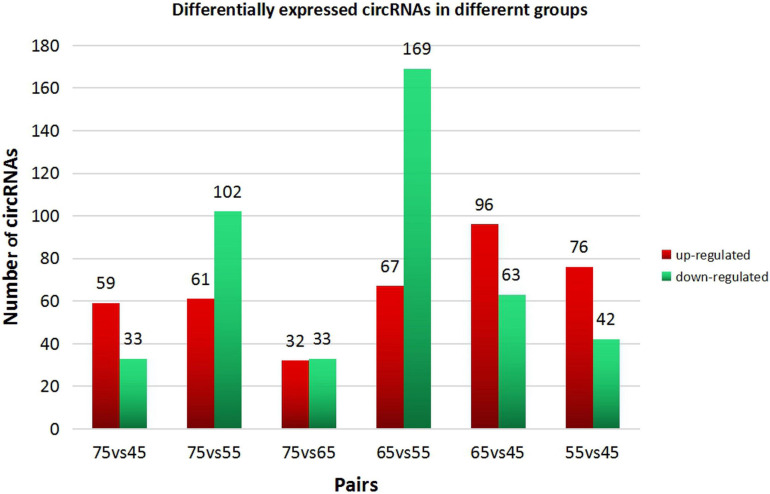
Differential expression of circRNAs in different groups. The red columns represent upregulated circRNAs, and the green columns represent downregulated circRNAs.

In order to explore further the regulatory role of circRNAs in the early development of cashmere goat hair follicles, we divided the four stages into six comparison groups and analyzed the differential expression by calculating the SRPBM value of circRNAs (Figures 3A,B). The results are as follows: d75vsd45, circRNA upregulated by 59 and downregulated by 33; d75vsd55, circRNA upregulated by 61 and downregulated by 102; d75vsd65, circRNA upregulated by 32 and downregulated by 33; d65vsd55, circRNA upregulated by 67 and downregulated by 169; d65vsd45, circRNA upregulated by 96 and downregulated by 63; and d55vsd45, circRNA upregulated by 76 and downregulated by 42 (Figure 4). By further exploring the law of differential expression of circRNA, it was found that the differential expression of circRNA of d65vsd55 was the greatest, while the differential expression of circRNA of d75vsd65 was the lowest (Figure 4 and Supplementary Figure 2).

### Validation of CircRNAs by qRT-PCR

**FIGURE 5:**
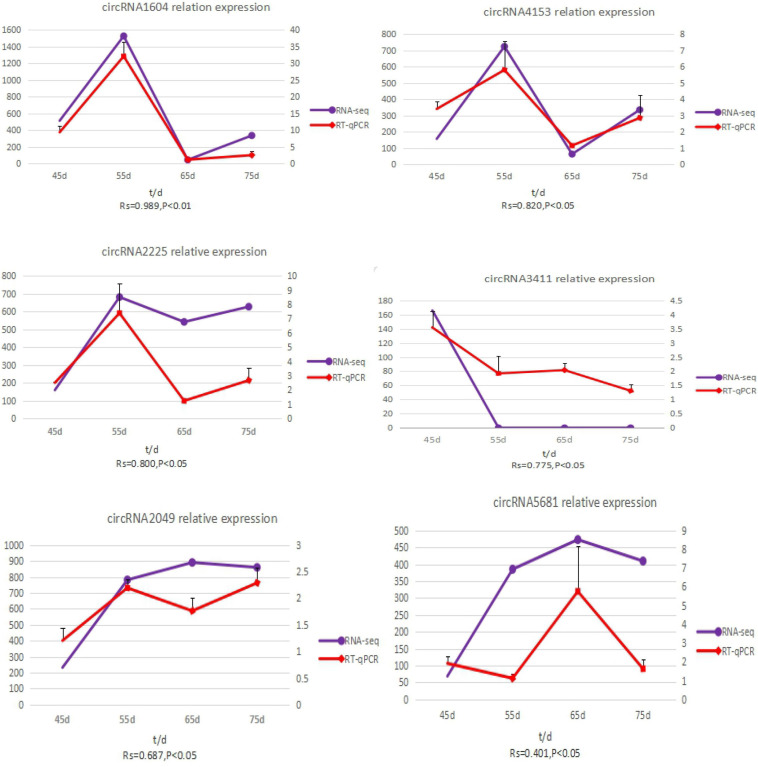
The expression quantity and expression trend of circRNA in different periods. A total of 0.8 < | Rs| < 1 indicates a strong correlation; 0.6 < | Rs| < 0.8 indicates a strong correlation; 0.4 < | Rs| < 0.6 indicates a moderate correlation; 0.2 < | Rs| < 0.4 indicates a weak correlation; 0 < | Rs| < 0.2 indicates no correlation; and the degree of proximity between | Rs| and 1 represents the degree of closeness and correlation between two variables.

To validate the accuracy of the circRNA sequencing results, the relative expression of six DE circRNAs (circRNA2049, circRNA3411, circRNA2225, circRNA5681, circRNA1604, and circRNA4351) (Supplementary Table 2), were measured by qRT-PCR (Figure 5). The qRT-PCR results were consistent with the transcriptome sequencing data.

### Gene Ontology and Kyoto Encyclopedia of Genes and Genomes Pathway Analysis of Host Genes

Gene Ontology analysis includes three domains describing the cellular and molecular roles of genes and gene products (BP, CC, and MF) (Harris et al., 2004). KEGG is a pathway database for the systematic analysis of gene function, linking genomic and functional information (Ogata et al., 1999). In order to explore the regulatory role of host genes of DE circRNAs in hair follicle genesis and development in cashmere goats, we analyzed the host genes of circRNA that were differentially expressed in different control groups by GO and KEGG pathway analyses. The results of GO enrichment showed that there was gene enrichment in the BPs related to the growth and development of hair follicles, such as hair follicle morphogenesis (GO:0031069), cell development (GO:0048468) MF including TGF beta receptor binding (GO:0005160), repressing transcription factor binding (GO:0070491), and CC including cell junction (GO:0030054), and transcription elongation factor complex (GO:0008023). A total of 54 pathways were significantly enriched in the six comparison groups. KEGG pathway analysis showed that there was gene enrichment in the Notch signaling pathway (ko04330), NF-κB signaling pathway (ko04064), PI3K-AKt signaling pathway (ko04151), and other signal pathways (Supplementary Figure 3). Therefore, the host genes corresponding to differentially expressed circRNAs may be involved in the process of hair follicle growth and development, and then play a regulatory role.

### Functional Analysis of CircRNA as an miRNA Sponge

**FIGURE 6:**
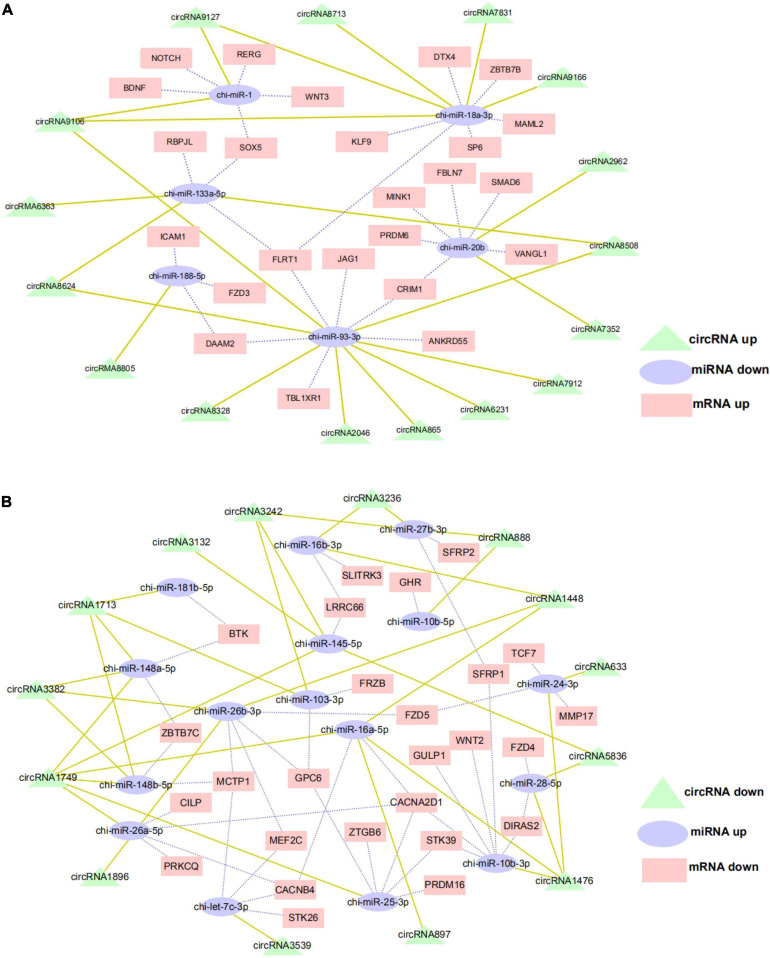
CircRNA–miRNA–mRNA regulatory network analysis in cashmere goat hair follicle. **(A)** Upregulated circRNA networks and **(B)** downregulated circRNAs networks for d75vsd45.

The ceRNA hypothesis is a new model for post transcriptional gene regulation. According to the hypothesis, the expression of designated miRNAs is reduced by ceRNA (Wang et al., 2019). To construct the ceRNA network of circRNA–miRNA–mRNA, we integrated our miRNA library data (unpublished data) and mRNA library data to analyze the miRNA binding sites in the circRNAs and mRNA using miRanda and Targetscan. We selected DE exon circRNA in d75vsd45 for construction of the ceRNA regulatory network. In the up–down–up regulation pattern, we predicted 46 circRNA–miRNA and 49 miRNA–mRNA interactions (Supplementary Table 3 and Figure 6A). As shown in Figure 6A, upregulated circRNA9106 may serve as a sponge for multiple miRNAs (chi-miR-1, chi-miR-18a-3p, and chi-miR-93-3p). Notably, three circRNAs, circRNA8058, circRNA6363, and circRNA8624 contained seed targets of chi-miR-133a-5p, which were identified by searching for miRNA target sites. Moreover, these miRNAs can downregulate the expression of their target genes (chi-miR-133a-5p was predicted to bind with *FLRT1*, *SOX5*, and RBPJL genes). We further constructed a down–up–down co-expression network using miRanda and Targetscan with a strict model, for which 56 circRNA–miRNA and 77 miRNA–mRNA interactions were predicted (Supplementary Table 4 and Figure 6B). In the down–up–down co-expression network, three downregulated circRNAs, circRNA3382, circRNA1448, and circRNA1896, contained seed targets of chi-miR-26b-3p. chi-miR-26b-3p was predicted to bind with multiple target genes, including *MCTP1*, *MEF2C*, *GPC6*, and *FZD5.* At the same time, circRNA3236 was predicted to have two target miRNAs (chi-miR-27b-3p and chi-miR-16b-3p).

### CircRNA3236 Binds to chi-miR-27b-3p and chi-miR-16b-3p

**FIGURE 7:**
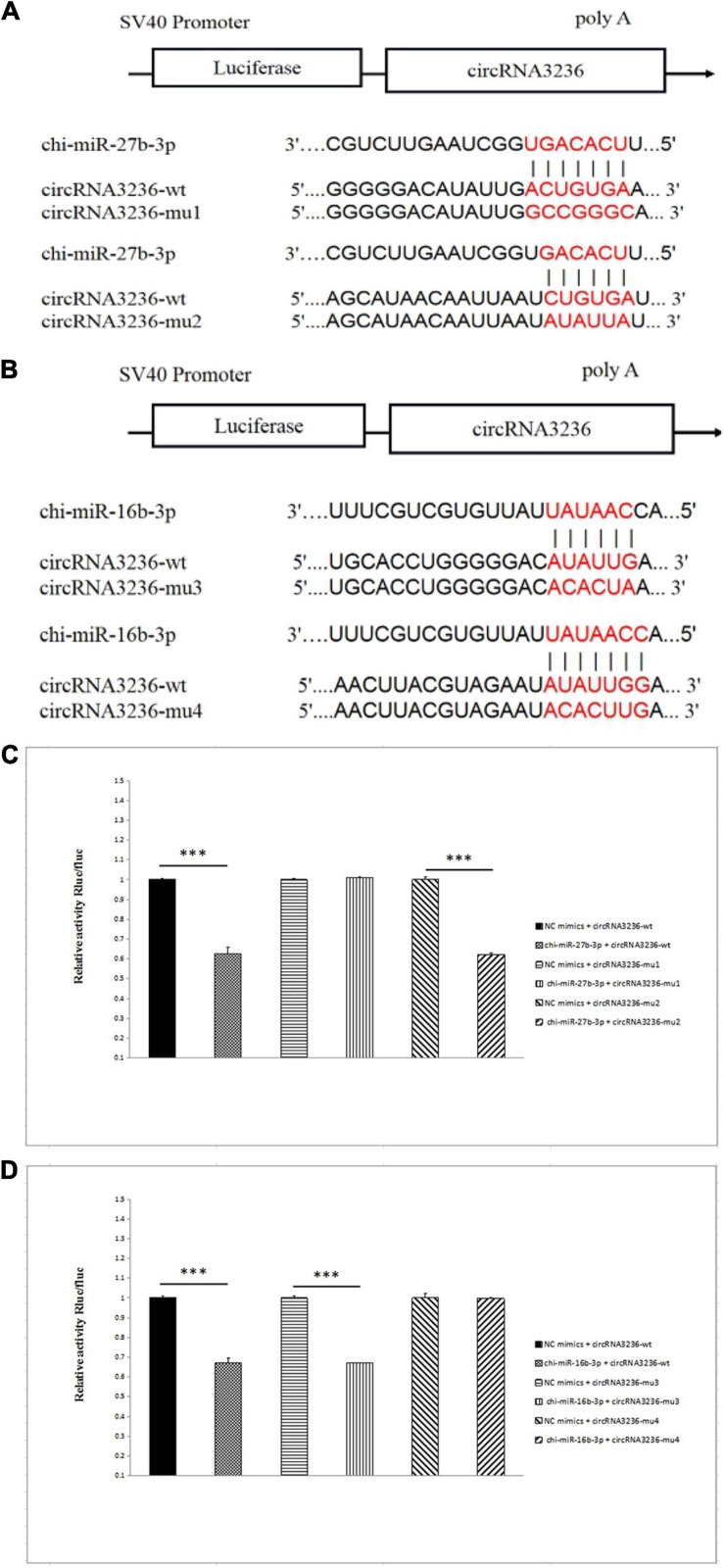
CircRNA3236 sponged with chi-miR-27b-3p andchi-miR-26a-3p. **(A)** The predicted binding site and mutated site of chi-miR-27b-3p in circRNA3236. **(B)** The predicted binding site and mutated site of chi-miR-16a-3p in circRNA3236. **(C)** Detection of interaction between circRNA3236 and chi-miR-27b-3p by dual luciferase reporter gene assay. **(D)** Detection of interaction between circRNA3236 and chi-miR-16b-3p by dual luciferase reporter gene assay. ^∗∗∗^*P* < 0.001 shows that the difference is extremely significant.

CircRNA3236 was downregulated in d75vsd45; Targetscan and miRanda software predicted that circRNA3236 was targeted to chi-miR-27b-3p and chi-miR-16b-3p, and there were two binding sites, respectively. Therefore, two mutant vectors were constructed to verify the specific binding sites (Figures 7A,B). The results showed that compared with the NC group, chi-miR-27b-3p and chi-miR-16b-3p significantly decreased the expression of luciferase in circRNA3236 WT (*P* < 0.001). It shows that there is a binding effect between the two in this experiment. After mu1 mutation, chi-miR-27b-3p failed to downregulate the expression of luciferase in circRNA3236-mut1 (*P* > 0.05), indicating that the mutation was successful. Mu1 is the binding site of chi-miR-27b-3p and circRNA3236-wt. After mu4 mutation, chi-miR-16b-3p failed to downregulate the expression of luciferase in circRNA236-mut4 (*P* > 0.05), indicating that the mutation was successful. Mu4 is the binding site of chi-miR-16b-3p and circRNA3236 (Figures 7C,D).

## Discussion

Studies have shown that circRNAs may affect biological function by regulating the level of linear mRNA expression (Kelly et al., 2015). In this experiment, the host genes of differential circRNAs were analyzed by GO and the KEGG pathway. The BP of GO enrichment includes hair follicle morphogenesis, hair follicle maturation, and cell growth; the pathway of KEGG enrichment includes the Notch signaling pathway and NF-kappa B signaling pathway. Previous studies have shown that there is a direct relationship between the Notch signal pathway and hair follicle morphogenesis (Lin et al., 2000), high expression of *Notch1* and *Notch2* can accelerate the formation of mouse hair substrate, and at the same time, it can inhibit the cells around the hair substrate to form substrate (Crowe et al., 1998). Ocu-miR-205 can promote the transition of Rex rabbit hair follicles from the growing stage to a degenerative quiescent stage by regulating the expression of related genes and proteins in Notch, BMP, and other signaling pathways, to change the hair density (Liu et al., 2020). Krieger used a mouse model to study the regulation of the NF-kappa B signaling pathway in the hair follicle cycle and found that the NF-kappa B signaling pathway is essential for the growth and activation of hair follicle stem cells (Krieger et al., 2018). The research of the NF-kappa B signaling pathway in mice showed that the NF-kappa B signaling pathway was activated downstream of EdaA1 and EDAR, thus, playing an important role in the development of hair follicles (Schmidt-Ullrich et al., 2006). Zhang et al. (2009) found that Wnt/β-catenin signaling transduction is a necessary signaling for NF-kappa B activation, and EDAR is the direct target gene of Wnt/β-catenin signaling pathway. The initial activation of the Wnt/β-catenin signaling pathway depends on the activity of EDA/EDAR/NF-kappa B in the prohair substrate of primary hair follicles. The complex interaction and interdependence of Wnt/ β-catenin and EDA/EDAR/NF-kappa B signaling pathways in the initiation and maintenance of primary hair follicle substrate were revealed (Zhang et al., 2009). Therefore, we speculate that circRNA may play a regulatory role in the primary stage of hair follicle development via the Notch signaling pathway and the NF-kappa B signaling pathway.

In the past few decades, there have been many studies on the regulatory role of miRNA and lncRNA in hair follicles (Sun et al., 2010; Liu et al., 2012; Yuan et al., 2013; Zhou, 2016, Wang et al., 2017; Zhou et al., 2018; Zhou, 2018). However, there is no report about circRNA related to hair follicle development. CircRNA is mostly located in the cytoplasm, and some circRNAs have MRE (a sequence recognized by miRNA), which can interact with miRNA and participate in molecular regulation as ceRNA. CeRNA was first proposed by the Pandolfi team at Harvard Medical School in *Cell* in 2011. It is pointed out that there are competitive endogenous RNA (ceRNA) molecules in cells. These ceRNA molecules (including lncRNA, circRNA, mRNA, pseudogenes, etc.) can compete through miRNA response elements (MRE) for a combination with the same miRNA in order to regulate each other’s expression levels (Salmena et al., 2011). In recent years, the involvement of circRNA as ceRNA in the regulation of biological life activities has become the focus of circRNA research. The research mainly focuses on human tumors and the regulation of life activities of some animals. In the field of human tumor research, researchers have constructed the circRNA–miRNA–mRNA regulatory network of liver cancer on the basis of high-throughput sequencing, which provided new insights that circRNA mediates the occurrence and development of liver cancer through a ceRNA mechanism (Zhao J. et al., 2019). It was found that circ_PUM1 could compete with miRNA-136, resulting in upregulation of *NOTCH3* expression, thus, promoting the occurrence and development of endometrial carcinoma (Zong et al., 2020). It was identified that hsa_circ_0000467 plays a regulatory role in gastric cancer by regulating the level of miRNA-326-3p, and that circRNA may be a potential diagnostic marker and therapeutic target in gastric cancer (He et al., 2020). It is verified that circFUT8 plays an inhibitory role in bladder cancer cells by targeting miRNA-570-3p/KLF10 (Mo et al., 2020). In non-human animal research, Zhang et al. (2020) found that circRNA-006258 regulates the growth and lactation of goat mammary epithelial cells through a circRNA-006258-miR-574-5p-EVI5L regulatory network. Wang et al. (2020) constructed a circRNA expression profile related to milk fat metabolism under heat stress and established the related ceRNA regulatory network. Li et al. (2020) analyzed the differentially expressed circRNA, between fast contractile muscle and slow contractile muscle of porcine skeletal muscle and established a ceRNA network by combining the differentially expressed circRNA with miRNA and mRNA databases, which was preliminarily verified by double luciferase. However, there are no reports on circRNA related to fetal hair follicle development in cashmere goats. In this study, a total of 21,784 circRNA were identified in four stages of fetal skin hair follicles of cashmere goats. The differentially expressed circRNA of each comparison group was screened, and the differential circRNAs were combined with miRNA and mRNA using the mode of upregulation–downregulation–upregulation or downregulation–upregulation–downregulation to construct the ceRNA network. Some circRNAs and differentially expressed circRNAs at different stages have been identified previously in the hair follicle development of Angora rabbits, but the total number of circRNAs, and differentially expressed circRNAs were greater, and a ceRNA network was established in this study. It is suggested that circRNAs may play an irreplaceable role in the development of hair follicles in cashmere goats (Zhao B.H. et al., 2019).

In order to explore further the functional mechanism of circRNA in the hair follicle development of cashmere goats, we predicted the miRNA targeted by circRNA and the target genes targeted by miRNA, which were differentially expressed by d75vsd45, and, thus, constructed the ceRNA network. Among them, 16 circRNAs targeted six miRNAs, and six miRNAs targeted 23 target genes in the upregulation–downregulation–upregulation model; 14 circRNAs targeted 16 miRNAs, and 16 miRNAs targeted 26 target genes in the downregulation–upregulation–downregulation model. We constructed circRNA2046-chi-miR-93-3p-FLRT1, circRNA2962-chi-miR-20b-SMAD6, circRNA3236-chi-miR-27b-3p-SFRP, circRNA3236-chi-miR-16b-3p-SLITRK3, circRNA1476-chi-miR-10b-3p-WNT2, and other signaling pathways. Among them, *SFRP1* (Hawkshaw et al., 2018), *WNT3* (Millar et al., 1999), *SMAD6* (Lv et al., 2019), and other genes are all involved in important pathways that have been related to hair follicle development in previous studies, including WNT, TGF-β, TNF, and other signal pathways. Therefore, we further speculate that circRNA, as ceRNA, may play a regulatory role in fetal skin hair follicles of cashmere goats through related genes such as WNT, TGF- β, and TNF. The results of the double luciferase experiment show that circRNA3236 and chi-miR-27b-3p, and circRNA3236 and chi-miR-16b-3p have a targeted binding relationship. It is suggested that the exon circRNA regulates gene expression by binding miRNA, and then regulates the growth and development of cashmere goat hair follicle. This is consistent with previous studies on the functional mechanism of circRNA.

## Conclusion

We constructed the expression profiling of circRNAs in the hair follicles of Inner Mongolia cashmere goats at different embryonic stages (45, 55, 65, and 75 days), and a total of 21,784 circRNA were identified. The results of GO and KEGG analysis of host genes showed that there may be some relationship between circRNA and its host genes, and circRNA may play a role in the BP of hair follicle growth and development in cashmere goats through the Notch signaling pathway and NF-kappa B signaling pathway. At the same time, the regulatory network of circRNA–miRNA–mRNA was constructed, and the interaction between 102 pairs of circRNA–miRNA and 126 pairs of miRNA–mRNA was studied. The results of the dual luciferase assay showed that circRNA3236 and chi-miR-27b-3p, and circRNA3236 and chi-miR-16b-3p have a targeted binding relationship. The specific binding sites were verified, which provides an important basis for exploring the molecular mechanism of circRNA as ceRNA in the morphogenesis and development of hair follicles, and also provides important information for studying the mechanism of action of circRNA in human hair follicles.

## Data Availability Statement

The RNA-Seq data were submitted to the SRA database under accession number (SRR13306949, SRR13306948, SRR13306947, SRR13306946, SRR13306945, SRR13306944, SRR13306943, SRR13306942, SRR13306941, SRR13306940, SRR13306939, SRR13306938). Additional data can be found in Supplementary Material.

## Ethics Statement

All fetal skin samples were collected in accordance with the International Guiding Principles for Biomedical Research Involving Animals and approved by the Special Committee on Scientific Research and Academic Ethics of Inner Mongolia Agricultural University, responsible for the approval of biomedical research ethics of Inner Mongolia Agricultural University (Approval No. [2020] 056). No specific permissions were required for these activities, and no endangered or protected species were involved.

## Author Contributions

FS, RM, YJZ, and JL conceived the idea and designed the study. FS, YW, ZXW, LL, and JP participated in the sample collection. FS, RM, EH, ZW, YR, and ZL performed the experiments. ZYW, YHZ, RW, and YJZ analyzed the data. FS and ZD wrote the draft. FS, ZD, YW, and YJZ finalized the manuscript. All authors read and approved the final manuscript.

## Conflict of Interest

The authors declare that the research was conducted in the absence of any commercial or financial relationships that could be construed as a potential conflict of interest.

## Footnotes

1 http://www.geneontology.org/

2 http://www.genome.jp/kegg/

3 http://www.targetscan.org/mamm_31/

4 http://www.microrna.org/microrna/home.do

## Supplementary Material

Supplementary Figure 1 Changeable process in hair follicle morphogenesis of the Inner Mongolian cashmere goat in the fetal period.

Supplementary Figure 2 Volcano plot of all DE circRNAs in six comparison groups.

Supplementary Figure 3 GO and KEGG enrichment analysis of host genes of DE circRNAs.

Supplementary Table 1 Primer information for qRT-PCR.

Supplementary Table 2 CircRNAs verified by qRT-PCR and related information.

Supplementary Table 3 CircRNA, miRNA, and mRNA in d45 vs d75 were differentially expressed in the up-down-up ceRNA regulatory network.

Supplementary Table 4 CircRNA, miRNA, and mRNA in d45 vs d75 were differentially expressed in the down-up-down ceRNA regulatory network.

## Abbreviations

CircRNA: circular RNA
ceRNA: competing endogenous RNA
qRT-PCR: quantitative RT-PCR.
