# Supplementary material for: Expression Profiling and Functional Analysis of Circular RNAs in Inner Mongolian Cashmere Goat Hair Follicles
Source: Front Genet. 2021 Jun 11;12:678825. doi: 10.3389/fgene.2021.678825 (PMC8226234; doi:10.3389/fgene.2021.678825)
Supplement: Supplementary Figure 1 — Changeable process in hair follicle morphogenesis of the Inner Mongolian cashmere goat in the fetal period. [file Data_Sheet_1.doc]

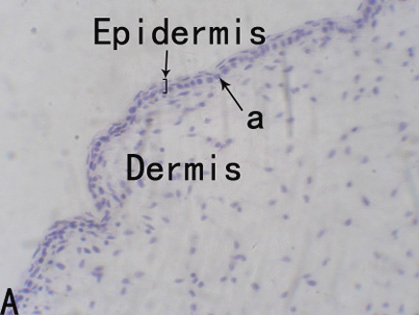

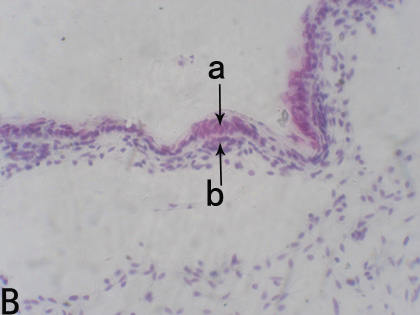

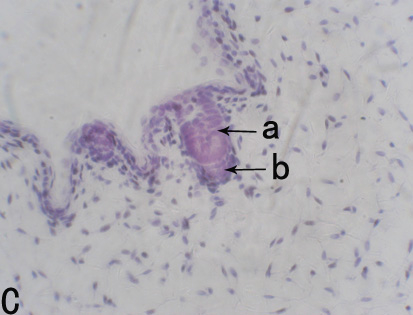

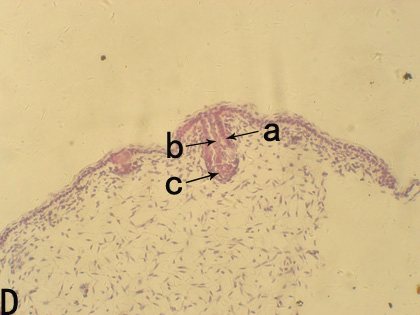

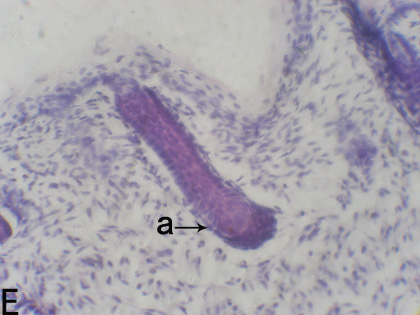

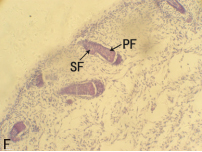


**Additional files 1：Figure S1** Changeable process hair follicle morphogenesis of the Inner Mongolian cashmere goat in the fetal period.

A. Midside of body at 55 days of fetal period（10×40）；epidermis. epidermis；dermis. dermis；a. pre-germ； B. Shoulder at 65 days of fetal period (10×40)；a. hair germ ；b. dermal fibroblasts；C. Midside of neck at 65 days of fetal period（10×40）；a. elongated hair germ ；b. cap-like condensation of dermal fibroblasts；D. Up-side of neck at 65 days of fetal period (10×20)；a. hair peg；b. columnar center keratinocytes；c. dermal fibroblasts formed a rounded dermal papilla；E. Poll at 75 days of fetal period（10×40）；a. connective tissue sheath；F.Poll at 75 days of fetal period（10×20）.
