# Supplementary material for: Expression Profiling and Functional Analysis of Circular RNAs in Inner Mongolian Cashmere Goat Hair Follicles
Source: Front Genet. 2021 Jun 11;12:678825. doi: 10.3389/fgene.2021.678825 (PMC8226234; doi:10.3389/fgene.2021.678825)
Supplement: Supplementary Figure 2 — Volcano plot of all DE circRNAs in six comparison groups. [file Data_Sheet_3.docx]

**
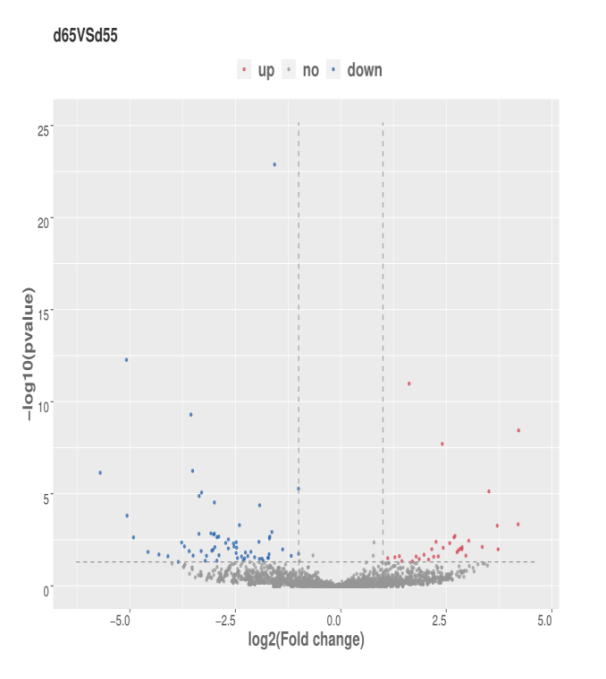

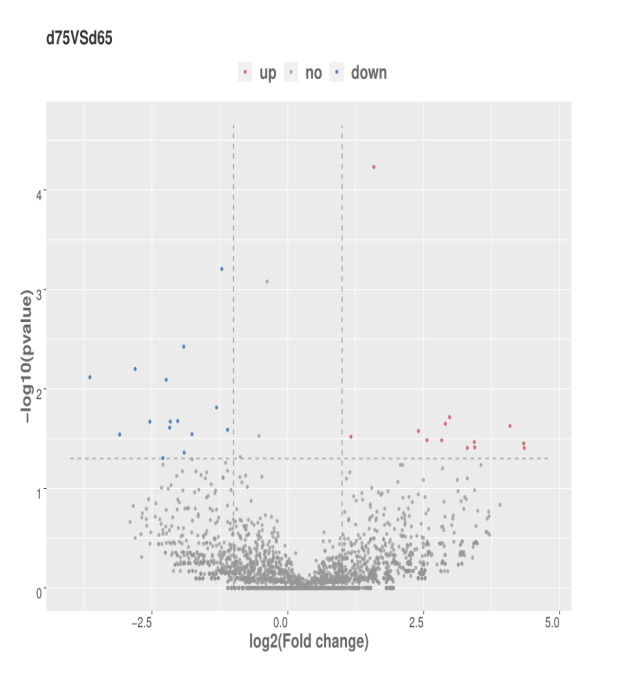

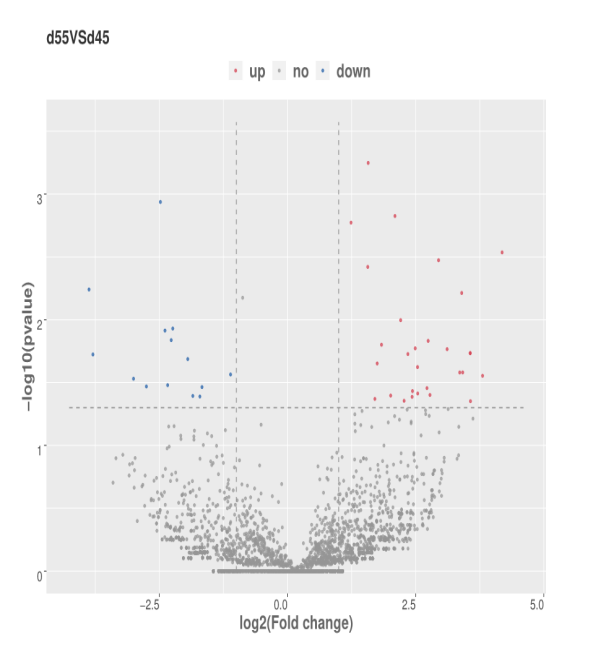

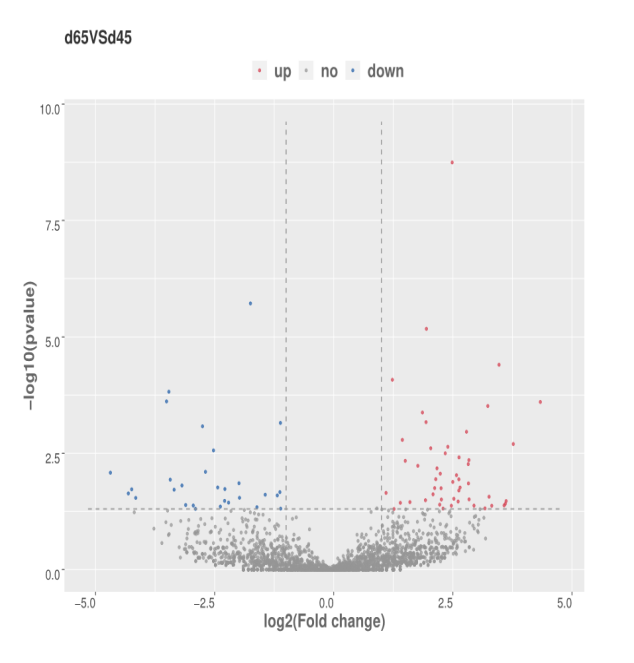

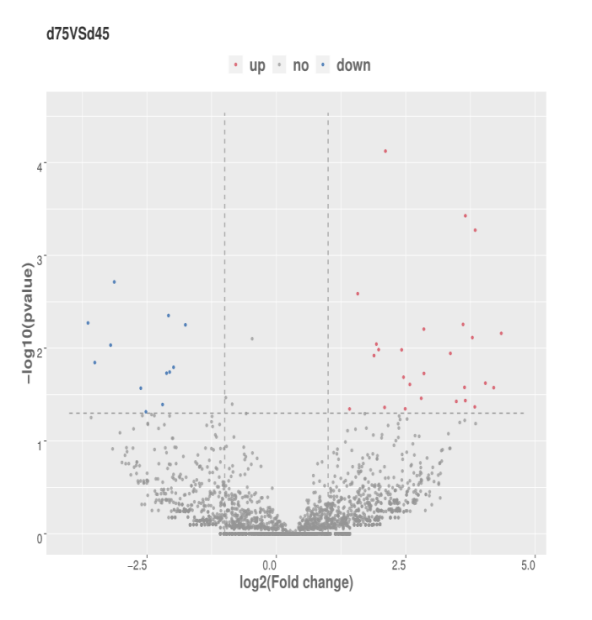

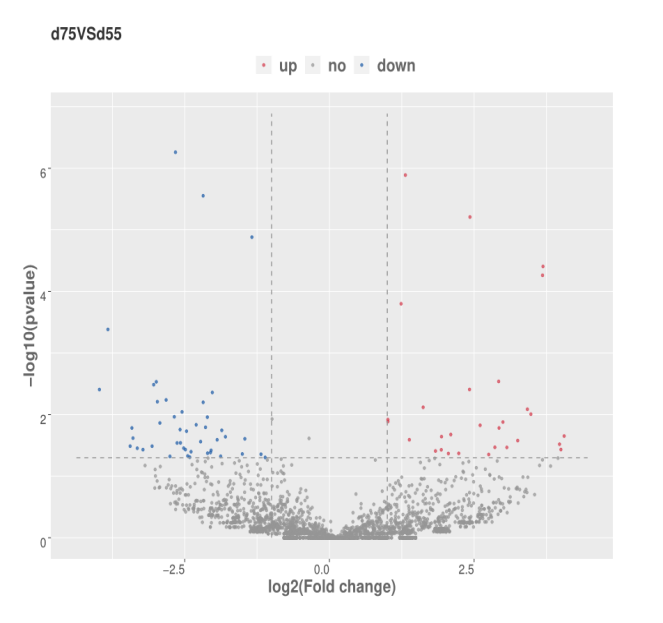
**

**Additional files 3:Figure S2** Volcano plot of all DE circRNAs in six comparison groups.Red represents upregulated circRNA and blue represents downregulated circRNA.
