# Supplementary figures and images for: Expression Profiling and Functional Analysis of Circular RNAs in Inner Mongolian Cashmere Goat Hair Follicles
Source: Front Genet. 2021 Jun 11;12:678825. doi: 10.3389/fgene.2021.678825 (PMC8226234; doi:10.3389/fgene.2021.678825)

**
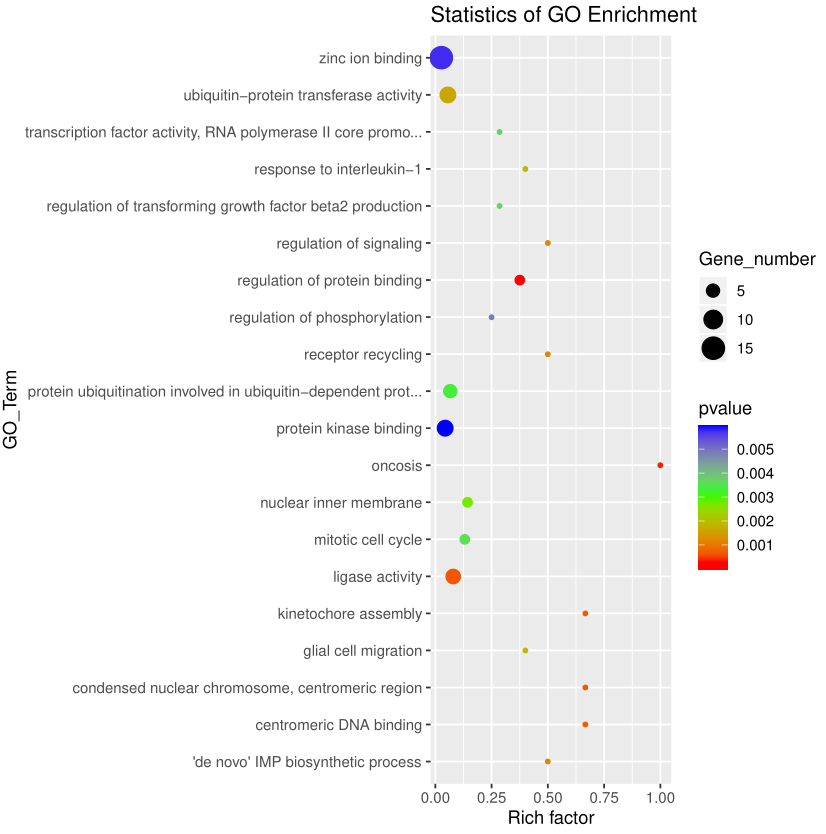

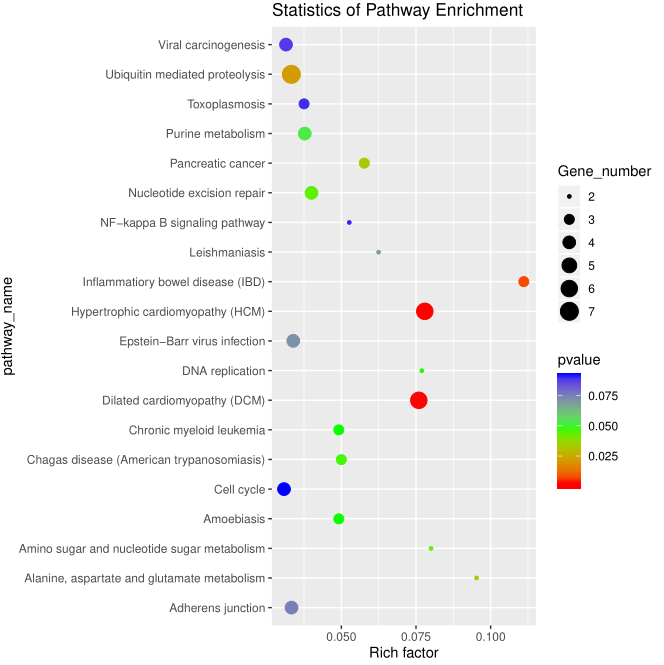

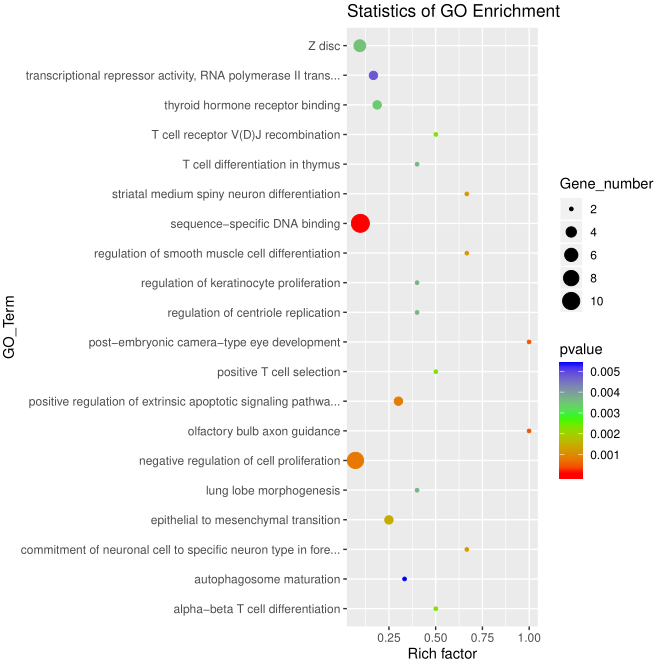

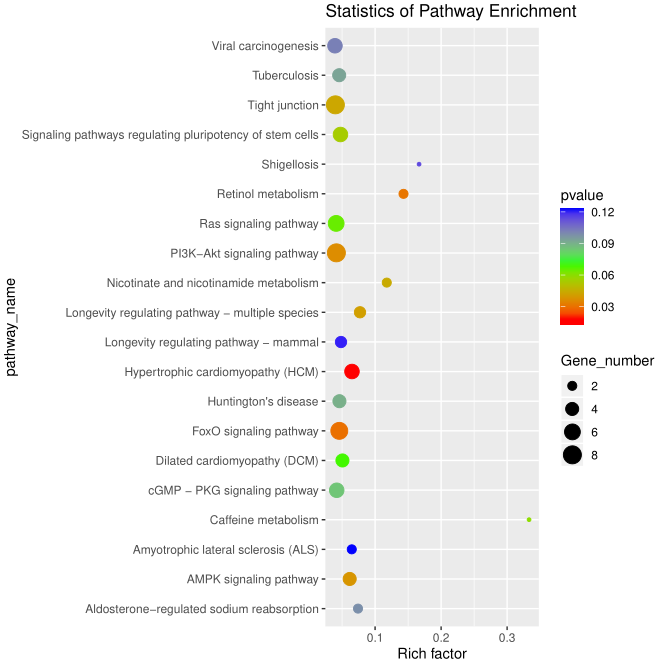

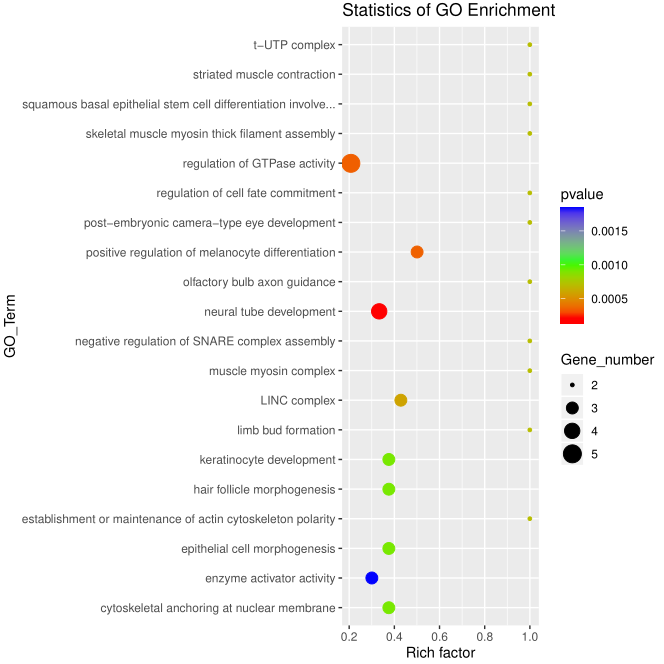

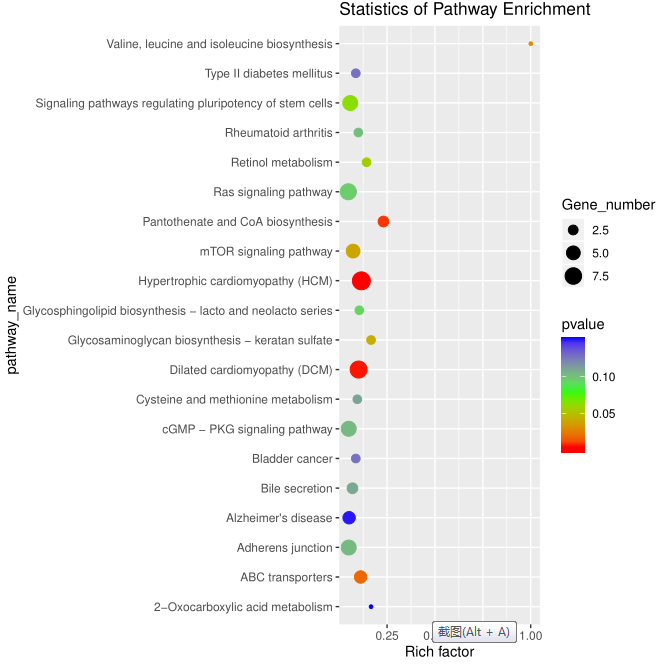
**

**
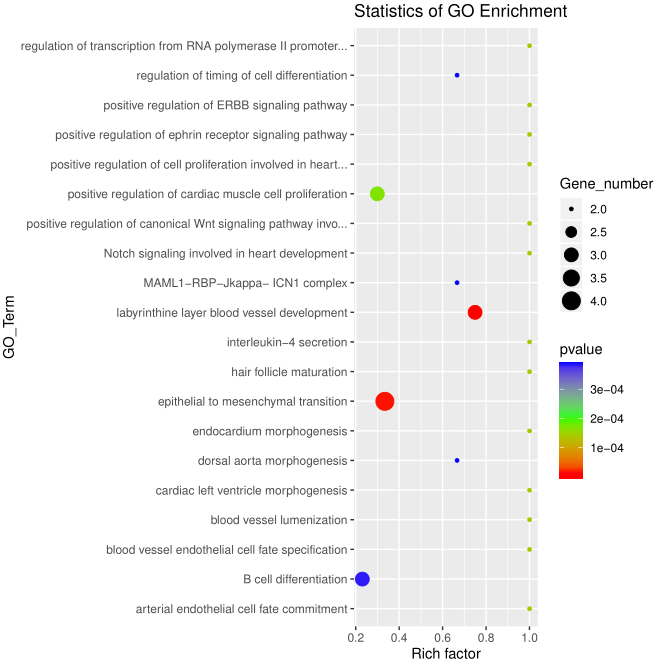

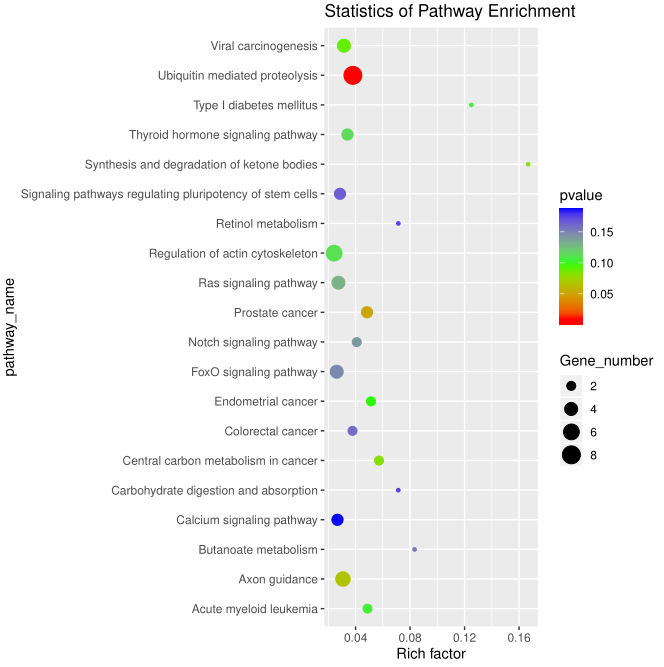
**

**
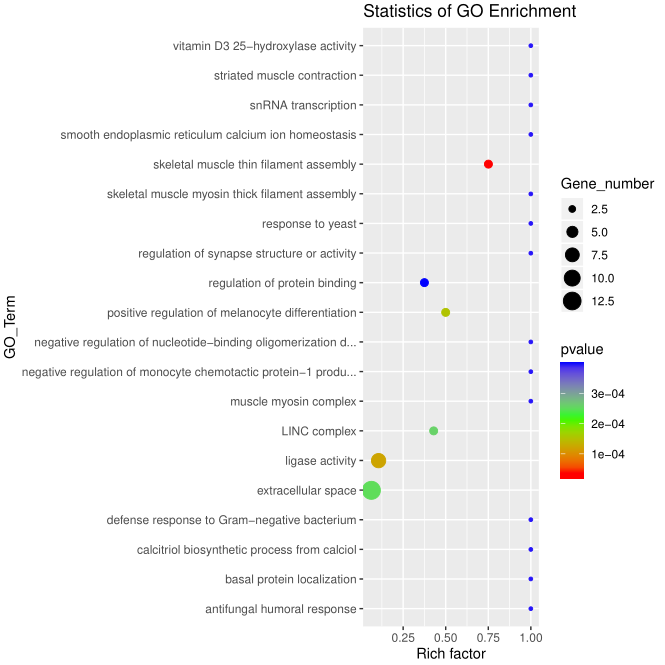

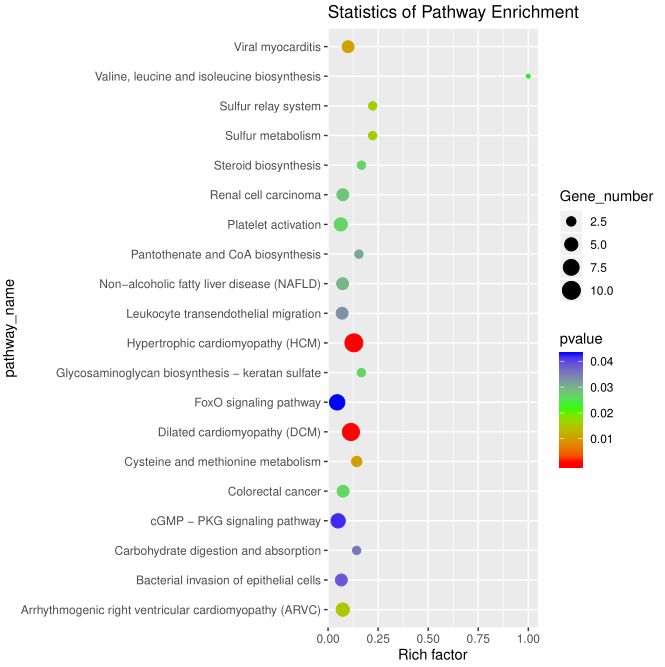
**

**
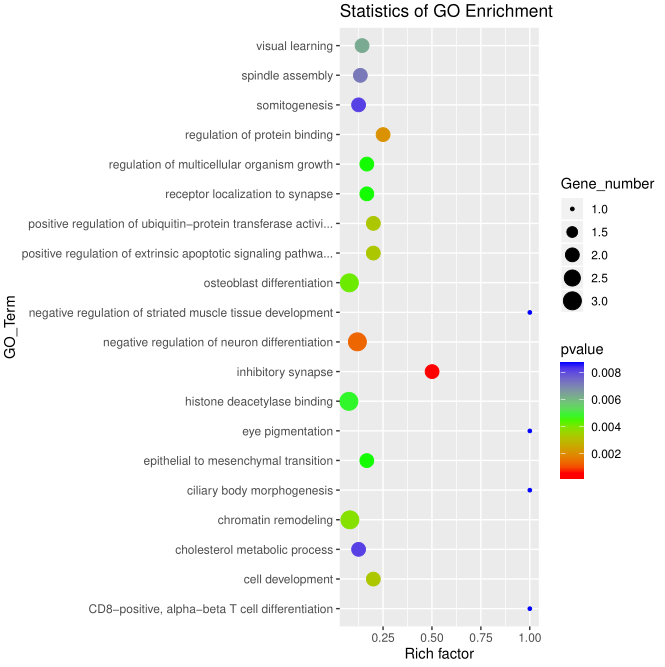

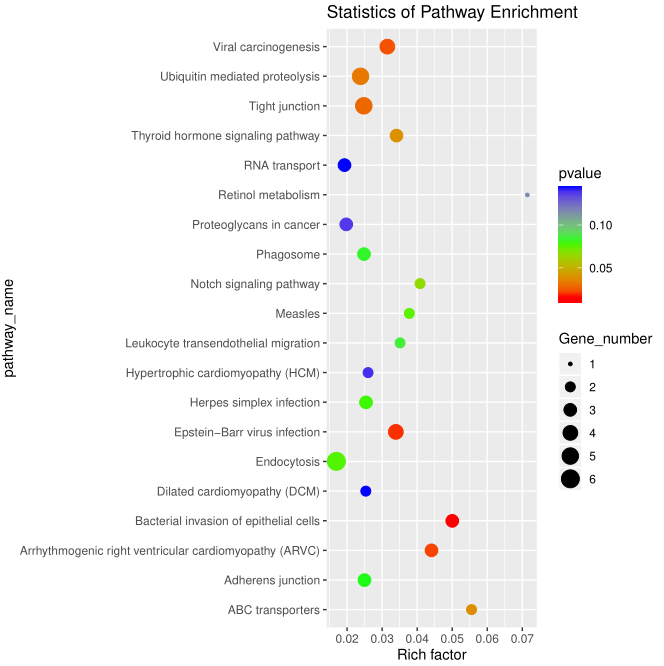
**

**Additional files5:Table S3** GO and KEGG enrichment analysis of host genes of DE circRNAs.

Supplement: Supplementary Figure 3 — GO and KEGG enrichment analysis of host genes of DE circRNAs. [file Data_Sheet_5.doc]
