# Supplementary material for: Expression Profiling and Functional Analysis of Circular RNAs in Inner Mongolian Cashmere Goat Hair Follicles
Source: Front Genet. 2021 Jun 11;12:678825. doi: 10.3389/fgene.2021.678825 (PMC8226234; doi:10.3389/fgene.2021.678825)
Supplement: Supplementary Table 1 — Primer information for qRT-PCR. [file Data_Sheet_2.doc]

Additional files 2:Table S1 Primer information for qRT-PCR

| Name | Primer | Sequene | primer length（bp） | Tm（℃） | Product  length（bp） |
| --- | --- | --- | --- | --- | --- |
| circRNA3411 | Forward | ACCTCATGATCTACAACCTGGA | 22 | 62 | 117 |
|  | Reverse | TCAAACAGAATCTCAAACGGCTG | 23 |  |  |
| circRNA2049 | Forward | | GCAACGATAGTTGGCAAGGTT | | --- | | 21 | 62 | 105 |
|  | Reverse | GGATAGCCTTCAATTAGCCATGTA | 24 |  |  |
| circRNA2225 | Forward | GTGTGTGCATCTTGTATTCGTGATC | 25 | 62 | 128 |
|  | Reverse | AACAGGCCATTCCAAAACAGACT | 22 |  |  |
| circRNA5681 | Forward | | TCGGTTCTCTCTCCTCCAGAACT |  |  | | --- | --- | --- | | 23 | 62 | 118 |
|  | Reverse | GTGAACAGCACGACAAACCTG | 21 |  |  |
| circRNA1604 | Forward | GCGCTATGGAAAGTCTGTAACGA | 23 | 60 | 119 |
|  | Reverse | CTTGTCTTGTTTTCGTTCTCTTCCA | 25 |  |  |
| circRNA4153 | Forward | CCGTCAATGACCTTGGGACA | 20 | 60 | 72 |
|  | Reverse | CTGGATTTTTGTAGATTTTGCCTTT | 25 |  |  |
| *β-actin* | Forward | GGCAGGTCATCACCATCGG | 19 | 60 | 158 |
|  | Reverse | CGTGTTGGCGTAGAGGTCTTT | 21 |  |  |
