# Supplementary material for: Expression Profiling and Functional Analysis of Circular RNAs in Inner Mongolian Cashmere Goat Hair Follicles
Source: Front Genet. 2021 Jun 11;12:678825. doi: 10.3389/fgene.2021.678825 (PMC8226234; doi:10.3389/fgene.2021.678825)
Supplement: Supplementary Table 2 — CircRNAs verified by qRT-PCR and related information. [file Data_Sheet_4.doc]

Additional files 4:Table S2 CircRNAs verified by qRT-PCR and its related information

| circRNA ID | circRNA  type | chr | circRNA_start | circRNA_end | Host gene symbol |
| --- | --- | --- | --- | --- | --- |
| circRNA2049 | exonic | NC_030833.1 | 16387152 | 16399896 | ATRNL1 |
| circRNA2225 | exonic | NC_030817.1 | 90131230 | 90146154 | SERINC5 |
| circRNA3411 | exonic | NC_030809.1 | 118098315 | 118098988 | 102173234 |
| circRNA5681 | exonic | NC_030835.1 | 32389704 | 32409788 | 102184877 |
| circRNA1604 | exonic | NC_030819.1 | 35520126 | 35552054 | LMO7 |
| circRNA4153 | exonic | NC_030812.1 | 64143969 | 64145981 | MYBPC1 |
